# Psychological impact of violence on male nurses in forensic units in Gauteng, South Africa

**DOI:** 10.4102/hsag.v28i0.2313

**Published:** 2023-08-29

**Authors:** Ntuthuko R. Thwala, Andile G. Mokoena-de Beer

**Affiliations:** 1 Department of Nursing Science, School of Health Care Sciences, Sefako Makgatho Health Sciences University, Pretoria, South Africa

**Keywords:** forensic units, male, psychological impact, psychiatric nurses, violence

## Abstract

**Background:**

Male psychiatric nurses are pivotal in providing treatment, care and rehabilitation to state patients admitted to forensic units. The nature of patients admitted in forensic units increase the likelihood violence for male psychiatric nurses. Substantial evidence suggests that a high incidence of violence in such units is linked to lack of security personnel amongst other factors, adding to the strain. Fewer studies adequately explored the psychological impact thereof specifically on male psychiatric nurses.

**Aim:**

To explore the psychological impact of violence on male nurses working in forensic units in Gauteng, South Africa, and the strategies used to deal with the impact of exposure to violence.

**Setting:**

The study was conducted at a mental health institution in the west of Tshwane Gauteng, South Africa.

**Methods:**

An exploratory, qualitative research design was used. In-depth interviews were used to collect data from 11 male psychiatric nurses. Data were analysed using thematic analysis.

**Results:**

Two main overarching themes emerged: (1) Traumatic experience and (2) Survival strategies to deal with the experience. The results suggest that exposure to violence has a debilitating psychological effect on male nurses, prompting them to utilise various ways to cope with the experiences. Psychological support and skills development could benefit male psychiatric nurses to manage the impact of violence adequately.

**Conclusion:**

Further research is recommended to explore the strategies to support male psychiatric nurses working in forensic units.

**Contribution:**

The study findings may be used to improve the psychological well-being of male psychiatric nurses working in forensic units in South Africa.

## Introduction

The treatment, care and rehabilitation of those admitted to forensic units are regulated by the *Criminal Procedure Act* No 51 of 1997, Section 77(6) (a) or 78(6) and *Mental Health Care Act* No 17 of 2022 (Republic of South Africa 2002:16). Forensic units in this study refer to maximum-security providing care to state patients. In accordance with these Acts, state patients are referred to as persons who have committed crimes, including rape, murder, malicious damage to property and other serious offences (Barr, Wynaden & Heslop 2019:889). The *Mental Health Care Act* also identifies psychiatric nurses as crucial players in providing care for those admitted to forensic units. However, while the Act clearly outlines the care, treatment and rehabilitation procedures, it does not cater for the psychological well-being of the psychiatric nurses involved. This paper explores the psychological impact of violence on male psychiatric nurses working in maximum-security forensic units at a mental health institution in Gauteng, South Africa.

Violence refers to the harmful behaviour an individual expresses towards others (Townsend & Morgan 2017). It is verbally expressed in the form of screaming, threatening and uttering abusive remarks. It can also be expressed nonverbally through physical assault or destruction of property, among others (Kneisl & Trigoboff 2014:744). The act of violence may have detrimental effects on the individuals experiencing it. In addition, while violence can occur in any setting, it is even more prevalent in mental health institutions and may be worse in maximum-security forensic units (Adeniyi & Puzi 2021; Mulaudzi et al. 2020). Male psychiatric nurses are key role players in caring for state patients in maximum-security forensic units. These state patients are potentially violent towards them, linked to the crimes they may have committed.

While working in mental health institutions can be stressful, it is assumed that working in maximum-security forensic units has added stress because of the nature of the persons being cared for in those units. Violence in forensic settings is widely reported, and thus, increased violence against nurses is noted (Ward 2013:284). This is supported by the findings from Dickens, Piccirillo and Alderman (2013:532), who confirmed that there are significantly higher rates of violence in forensic settings occurring at 47.7% than in acute mental health settings at 26.2%, thereby, intensifying the stress levels in these units. Hasan and Tumah (2019:153) concur that stress levels tend to be heightened in these contexts as state patients demonstrate violence towards psychiatric nurses. The experience may affect the psychiatric nurses physically and emotionally, resulting in low rates of job satisfaction, elevated stress levels, increased sick leave, and a desire among employees to quit their job (Dickens et al. 2013:532). Consequently, violence may hinder the therapeutic relationship between male psychiatric nurses and state patients.

Male psychiatric nurses are accustomed to stating that patients are aggressive and potentially dangerous and are socialised to accept this behaviour (Hasan & Tumah 2019:156). Violence is typically directed towards these psychiatric nurses through nonverbal threats, such as pounding on the staff office door, random throwing of objects, destruction of property and verbal threats that frighten them (Wik 2018). According to Lantta et al. (2016:1), psychiatric nurses working in psychiatric units are 40% – 70% more likely to experience physical violence than non-physical violence. It is also noted that psychiatric nurses working in mental health institutions are exposed to violence at least once during their careers. A systematic review conducted by Odes et al. (2021:34) revealed a 25% – 85% chance of violence being directed towards healthcare workers in the United States inpatient psychiatric hospitals each year. Similarly, a study by Jang, Son and Lee (2022:454) reported a prevalence rate of 11.4% – 97%. This could be worse for male psychiatric nurses in maximum-security forensic units as they work with state patients who are potentially perceived as dangerous.

Violence against male psychiatric nurses is a global concern as they spend significant time with these patients. They are more exposed to all forms of injuries while managing violent patients (Bekelepi, Martin & Chipps 2015:160). Thus, maximum-security forensic units are viewed as a tense and threatening environment for male psychiatric nurses and other state patients because of the patients’ violent nature (Kumpula & Ekstand 2013:65). While most state patients cannot control their aggression, the scarcity of resources including security personnel in maximum-security forensic units, inadequate training and poor adherence to safety protocols may make it difficult for male psychiatric nurses to deal with the state patients, contributing to nurses feeling that their safety is being undermined (Mulaudzi et al. 2020:3; Wik 2018:3). Mulaudzi et al. (2020:3) reported that working in specialised mental health institutions is perceived as a challenge because of inadequate safety measures to manage the violent nature of the patients in such settings.

In addition to the prevalent violence, the psychiatric nurses working in maximum-security forensic units do not possess the necessary forensic nursing skills, which may place them at a greater risk (Barr et al. 2019:894; Harris, Happell & Manias 2015:134). There is often a lack of security personnel in the maximum-security forensic units, adding to the strain of working in such units. This has workload implications for the male psychiatric nurses who must address aggressive incidents in addition to their work. The situation can consequently have an overwhelming physical and psychological impact (Bekelepi et al. 2015:161). A fair number of qualitative studies on the experience of violence in maximum-security forensic units exist (Dickens et al. 2013:532–544; Harris et al. 2015:130–138). While most studies have reported on the incidence and physical impact of violence in forensic units, fewer studies have adequately explored the psychological impact thereof (Bekelepi & Martin 2022:3; Lantta et al. 2016:2; Odes et al. 2021:28). There has been no primary focus on male psychiatric nurses and violence experienced in maximum-security forensic units in South Africa. The psychological impact resulting from the exposure may not be observed because of its subjective nature. Odes et al. (2021:28) posit that exposure to violence in such settings has varied emotional and psychological consequences. The psychological impact could manifest in high-stress levels, ineffective ways of managing stress and primarily poor mental health (Hasan & Tumah 2019:158; Jang et al. 2022:454; Odes et al. 2021:28).

Adverse work-related outcomes have also been reported, such as an inability to perform work roles, which could ultimately further threaten the safety of others (Hasan & Tumah 2019:154). A study by Joubert and Bhagwan (2018:54) suggests that psychiatric nurses struggle to deal with disruptive behaviour, specifically violence perpetrated by state patients, consequently making it difficult for them to cope with their duties. Psychiatric nurses ultimately require effective coping mechanisms to deal with their exposure to violence in maximum-security forensic units (Harris et al. 2015:131).

While exposure to violence has detrimental effects on psychiatric nurses and the therapeutic relationship, there is a need to understand the psychological impact violence has on the male psychiatric nurses (Jang et al. 2022:460; Joubert & Bhagwan 2018:49; Odes et al. 2021:28). Little is known in the context of South African male psychiatric nurses about the extent of these effects because of violence. The studies conducted in South Africa reported non-availability of resources as one of the contributing factors to the violence experienced in maximum-security forensic units (Joubert & Bhagwan 2018:54; Mulaudzi et al. 2020:4). There is thus a need to explore further the psychological impact of violence and the strategies used by male psychiatric nurses to cope with exposure to violence in resource-constrained maximum-security forensic units at a mental health institution in Tshwane.

## Aim

The study aimed to explore the psychological impact of violence on male psychiatric nurses in a mental health institution’s maximum-security forensic units in South Africa and the strategies used to deal with the experience.

## Research design

An exploratory qualitative research design using a phenomenological approach was used to explore the psychological impact of violence on male psychiatric nurses and the strategies used to cope with their experience in maximum-security forensic units in Gauteng, South Africa. An exploratory qualitative research design is particularly suitable for this study as it seeks to understand the psychological impact of violence from the perspective of male psychiatric nurses in South Africa. Little is known about their experience (Babbie 2021:91). The study was guided by the constructivist theory which is based on the philosophical orientation that reality and the way it is understood is subject to the context and the meaning created thereof (Singh et al. 2022:24). Constructivism is central to human experience, thus relevant for this study as the researcher had to first understand how the male psychiatric nurses experience violence in their context (maximum-security forensic units) in order to understand the psychological impact thereof.

## Research setting

This study was conducted at a mental health institution in the west of Tshwane, Gauteng, South Africa. There are only two maximum-security forensic units at the selected mental health institution referred to as unit A and B in this paper. These are strictly male units, thus no female personnel are allocated to work in these units because of the safety risk. Unit A has a 40-bed carrying capacity with 13 professional nurses, 4 enrolled nurses, 4 auxiliary nurses and 5 social auxiliary workers. The social auxiliary workers are there to assist nurses with access control of the maximum-security forensic units and promotion of a safe environment for both staff, other external stakeholders and other mental health care users in the unit. Unit B had a carrying capacity of 30 beds and has recently been reduced to 25 beds due to resource constraints. There are 9 professional nurses, 4 enrolled nurses and 4 auxiliary nurses in unit B with no social auxiliary workers. The institution was chosen, as it is one of the few mental health institutions in Gauteng, South Africa that provides forensic mental health services.

### Population and sampling

The male psychiatric nurses were purposively selected (Grove & Gray 2021:429), based on their known experience in the forensic units that identified them as potential study participants. The applied inclusion criteria were male psychiatric nurses who are exposed to violence while working in forensic units, must have exclusively worked in forensic units at the time of exposure to violence, are capable of giving consent to participate and are able to speak English. Thus, 11 male psychiatric nurses participated in the study, and the sample size was therefore guided by the principle of data saturation, a point where no new information is elicited (Polit & Beck 2021:177).

### Recruitment process

The primary author gained access to the participants following approval from the Chief Executive Officer of the mental health institution to collect data. The participants were approached directly after being identified as potential participants of the study. Appointments were made with the male psychiatric nurses who voluntarily agreed to participate in this study for the purpose of obtaining informed consent and collect data.

### Data collection

In-depth individual interviews were conducted to collect data at the participants’ workplaces in an allocated office within the forensic units. The first author conducted all interviews from April 2021 to July 2021; these were triangulated with field notes and observations. The field notes and observations were recorded using a diary detailing events and the participants’ reaction during the interviews (Polit & Beck 2018:207; Singh et al. 2022:196). The interviews lasted for approximately 30 min–60 min, were audio-recorded with participants’ consent and transcribed verbatim. No one else was present during the interview sessions.

A central question posed to each participant was: *What is the effect of violence on you as a male psychiatric nurse working in maximum-security forensic units?* Prompts were also used as open-ended questions to expand on the participants’ descriptions. The prompts were guided by how the participants responded to the questions. Examples of the prompts are:

- ‘How does this experience impact you?’;
- ‘How does being exposed to violence in forensic units affect your work?’;
- ‘How have you been dealing with the exposure to violence in forensic units?’;
- ‘How did you cope following the exposure to violence in the forensic unit?’; and
- ‘In your view, what can be done to help you deal with the impact of being exposed to violence in forensic units?’.

### Data analysis

Data were analysed using thematic analysis. This process analysed data by identifying common themes, topics, ideas and patterns of meaning that emerge from the data (Nowell et al. 2017). The process involved preparing the data; reading and memoing ideas, describing and classifying codes into themes; developing and assessing interpretations and representing and reporting the findings (Creswell & Poth 2018:189). Both authors conducted a preliminary analysis of the transcribed interviews independently from the coder. A consensus discussion was then arranged with the coder to agree on and finalise the themes. The themes were grouped into broader and more abstract categories of meaning; see Table 2 (LoBiondo-Wood & Haber 2022:102).

### Ethical considerations

Ethical approval was received from Sefako Makgatho Health Sciences University Research Ethics Committee (SMUREC) (No. SMREC/H/13/328/ 2020: PG) and permission to conduct the study was obtained from the institution’s Chief Executive Officer inline with the Standard Operating Procedure as set by Gauteng Department of Health.

In addition, the researchers adhered to the ethical principle of autonomy, by allowing the participants to make an informed decision about their participation thus obtained informed consent (LoBiondo-Wood & Haber 2022:247). Anonymity was maintained by not using the male psychiatric nurses’ real names when conducting the interviews, instead assigning them numerical codes. To maintain confidentiality, no information provided by the male psychiatric nurses was linked to them. Participants were not harmed by ensuring that they do not experience humiliation during the interviews. All participants were treated fairly and without bias or discrimination (LoBiondo-Wood & Haber 2022:246).

### Measures to ensure trustworthiness

The level of trust that researchers have in their data and analysis is referred to as trustworthiness (Polit & Beck 2021:276). To ensure *credibility*, data saturation was reached with nine participants; however, two more interviews were conducted to ascertain saturation, thus prolonging engagement with the participants. The researcher also returned to the participants to share the interpretation of findings and query accuracy from the perspective of the male psychiatric nurses who have been exposed to violence in forensic units (Singh et al. 2022:340). This was done through setting up appointments with the interviewed participants and sharing with them the transcribed and analysed data to verify whether it reflects their description. The researcher ensured the study’s *transferability* by providing a well-documented account of all the processes including a dense description of participants and the setting (Bertram & Christiansen 2018a, 2018b). *Confirmability* of the study was achieved through using several data collection methods such as interviews, field notes and observations. An independent coder was used to analyse data after the findings of both the researcher and the independent coder were compared to avoid researcher bias. *Dependability* was ensured by providing a detailed description of the research methodology used, providing a detailed description of the data analysis method and conducting a thorough review of the literature.

## Results

Below is a summary of the demographics of the participants as well as themes reflecting the male psychiatric nurses’ description of the psychological impact of violence.

### Description of demographics

Sufficient information was obtained from 11 male psychiatric nurses. The participants were enrolled (four = 36.4%), professional (four = 36.4%) and advanced psychiatric nurses (three = 27.2%). Their ages ranged from 27 to 59 years, and they had between 3 and 12 years of experience in a forensic unit. On average, participants had 5 years of experience (see Table 1).

**TABLE 1:** Participants’ demographic characteristics (***N*** = 11).

Participant no	Age (years)	Level of education	Rank	Years of experience in the field	Years of experience in the acute forensic unit
Participant 1	29	Master’s Degree	Advanced psychiatric nurse	5	3
Participant 2	31	Honours Degree	Advanced psychiatric nurse	7	4
Participant 3	27	Bachelor’s Degree	Professional nurse	4	3
Participant 4	33	Bachelor’s Degree	Professional nurse	8	4
Participant 5	38	Higher certificate in Nursing	Enrolled nurse	9	3
Participant 6	35	Higher certificate in Nursing	Enrolled nurse	8	5
Participant 7	33	Higher certificate in Nursing	Enrolled nurse	7	3
Participant 8	57	Bachelor’s Degree	Professional nurse	23	12
Participant 9	41	Higher certificate in Nursing	Enrolled nurse	11	7
Participant 10	45	Honours Degree	Advanced psychiatric nurse	13	6
Participant 11	59	Honours Degree	Professional nurse	19	8

*Source:* Adapted from Mokoena, A.G., 2012, ‘Facilitation of the mental health of couples in a relationship where one is challenged with mental illness’, pp. 33–34, Minor dissertation, University of Johannesburg, Auckland Park, M.Cur Psychiatric Nursing.

### Presentation of themes

Two main overarching themes emerged as: (1) traumatic experience and (2) survival strategies to deal with the experience (see Table 2). From these results, it is noted that the experiences have a psychological impact on male psychiatric nurses described as traumatic leading them to resort to enforcing tight safety measures to minimise the risk without necessarily dealing with the psychological impact thereof. The male psychiatric nurses made recommendations. Each theme is supported with multiple quotations from male psychiatric nurses to support and provide an understanding of their experience.

**TABLE 2:** Male psychiatric nurses’ description of being exposed to violence.

Theme	Main themes	Sub-themes
Theme 1	Male psychiatric nurses describe the psychological impact of violence as traumatic	Debilitating emotional effect Fear following exposure to violence Resultant lack of motivation to work and poor work attendance
Theme 2	Male psychiatric nurses describe the survival strategies used to deal with the exposure to violence.	Accepting violence as part of work Reinforcing safety measures Continuous supervision

#### Theme 1: Male psychiatric nurses experienced their exposure to violence as traumatic

The psychiatric nurses in forensic units experience different forms of violence such as physical, emotional and verbal aggression. The male psychiatric nurses in this study experienced both verbal and physical forms of violence. The male psychiatric nurses described the experience of violence being directed towards them as traumatic. This trauma was linked to the nature of crimes the state patients committed and the participants’ views towards the state patients. One of the participants described his traumatic experience as follows:

‘Traumatic, I mean seeing somebody having killed the whole family, and then you deal with that person. It’s very traumatic, knowing that you’re dealing with a hardcore criminal or a person who is mentally ill to do such things … being exposed to this violence.’ (Participant 1, 29-year-old advanced psychiatric nurse)

The participants further described their experiences as debilitating, affecting them emotionally. As a result, they demonstrated ineffective coping mechanisms as they continued to live in fear. This manifested in psychological patterns linked to mental health disorders:

‘It affects the other colleagues because there no resources to work with, so most of them fall into depression.’ (Participant 3, 27-year-old professional nurse)

‘Sometimes you don’t sleep at night it affects you physically, you lose weight.’ (Participant 7, 33-year-old enrolled nurse)

The exposure to violence impacted negatively on the male psychiatric nurses’ motivation to work as well as work attendance because of fear following the incidents. Some chose to absent themselves from work to avoid being confronted with violence and dangerous situations.

‘You don’t come to work because you’re afraid that your life is in danger because of such patients.’ (Participant 7, 33-year-old enrolled nurse)

However, increased absenteeism affected the remaining psychiatric nurses. Ultimately, the unpredictable nature of the state patients kept the male psychiatric nurses on edge. They remained fearful when caring for the state patients while their workload increased, resulting in an inability to complete their daily tasks. This left them feeling helpless:

‘You can’t complete your tasks, at least not always…’ (Participant 3, 27-year-old professional nurse)

‘Like uhh … it’s not safe at all because these patients are unpredictable, anything is possible, so you have to be alert at all times.’ (Participant 4, 33-year-old professional nurse)

‘It is so painful to see a patient being violent because sometimes you cannot even do anything with the violence because of shortage of manpower and sometimes you get injured physically.’ (Participant 7, 33-year-old enrolled nurse)

The male psychiatric nurses’ safety became compromised, further perpetuating their experience of trauma because of violence. Some of the participants reflected on this experience and explained:

‘I don’t feel safe, but as day in and out as I’m learning, I’m getting used to it, but I don’t feel safe at all, my safety has been compromised.’ (Participant 2, 31-year-old advanced psychiatric nurse)

‘So, me being here means I’m also not safe. But then even if I’m not safe I don’t have to show them that I’m scared.’ (Participant 4, 33-year-old professional nurse)

‘To be honest it’s very scary and you need to always watch your back because anything can happen anytime.’ (Participant 8, 57-year-old professional nurse)

#### Theme 2: Survival strategies to deal with the experience

Through this experience, the male psychiatric nurses resorted to accepting violence as part of their work in order to cope with the effects. These were mainly used as survival strategies in order to cope with being exposed to violence. Even though it is an expected responsibility of the psychiatric nurses in maximum-security units to implement such measures, the male psychiatric nurses implemented these measures with more vigilance to ensure their own safety than they did before the exposure. They learnt to live with the situation and accepted this phenomenon as part of their work environment. The statements below support this view:

‘Eh … I ended up accepting the situation as it is …’ (Participant 1, 29-year-old advanced psychiatric nurse)

‘What I can say is that I think we are adjusting as time goes on.’ (Participant 2, 31-year-old advanced psychiatric nurse)

‘The experience at the beginning is scary but as time goes by you get used to it and you learn to manage them.’ (Participant 6, 35-year-old enrolled nurse)

‘I don’t feel safe at all, but as day in and out I’m learning, I’m getting used to it.’ (Participant 9, 41-year-old enrolled nurse)

Furthermore, the male psychiatric nurses revealed that they found themselves enforcing safety protocols by tightening security and safety measures to prevent further incidents of violence. Some of these are roles that could be fulfilled by security personnel. The number of security personnel in these units is not enough. Thus, the male psychiatric nurses engage in activities that are directly linked to security personnel to minimise the risk of violence towards them. The participants explained:

‘The mental health care users (state patients), we search them. And then we normally count them on a daily basis … we search like the spoons they use … to sharpen the spoons to make weapons, so we count the spoons. We follow all security protocols. Then we can avoid any harm.’ (Participant 1, 29-year-old advanced psychiatric nurse)

‘Anything to them, they can use it as a weapon, so it is not safe, but we do search them.’ (Participant 9, 41-year-old enrolled nurse)

‘Even though we don’t have security guards in the premises but at least we do random searches and all that just to be safe.’ (Participant 10, 45-year-old advanced psychiatric nurse)

Continuous supervision of state patients was an integral part of the male psychiatric nurses’ role. This enabled them to be vigilant and ensure that all state patients were within a close vicinity so they could be closely observed. The participants revealed:

‘So, we must observe them as well as their behaviours so we dealing with a person who is unpredictable, and you don’t know that person.’ (Participant 5, 38-year-old enrolled nurse)

‘Must always be vigilant at all times, and then know each and every person’s personality.’ (Participant 11, 59-year-old professional nurse)

‘But anyhow, you must always be like … watch out, looking at the patients, or what are they doing, or what are they formulating.’ (Participant 2, 31-year-old advanced psychiatric nurse)

‘Always facing the patient’s direction to see where the patient is sitting at and what he is doing so that you must not be caught by surprise.’ (Participant 6, 35-year-old enrolled nurse)

Over and above enforcing security and safety protocols, the use of psychiatric nursing skills has assisted the male psychiatric nurses to deescalate during violent acts by the state patients. The use of these skills is essential in any psychiatric unit. These skills are even more critical in maximum-security forensic units as they become a useful resource to manage acts of violence in the workspace. The skills include intervening or understanding state patients’ perspectives. Some participants also mentioned effective communication skills help to defuse state patients’ violence. The participants elaborated:

‘You have to sit with the patient and intervene, hear their sides of the story and understand what brought them in hospital so it becomes easier, and the patient also learn to have trust in you.’ (Participant 9, 41-year-old enrolled nurse)

‘But when you are used, you know how to manage how to negotiate and talk to them when they are aggressive, the way you talk with him.’ (Participant 5, 38-year-old enrolled nurse)

‘Most of the time when you’re working with them, you must mind your words, check the environment properly each and every day, they are dangerous.’ (Participant 10, 45-year-old advanced psychiatric nurse)

## Discussion

This study found that male psychiatric nurses experience trauma from exposure to state patients’ violence. The phenomenon manifested in physical scars and debilitating psychological impact on male psychiatric nurses. Participants confirmed that they were exposed to psychological stress, evident when they displayed maladaptive patterns in the workplace, presenting as heightened fear and helplessness. Other forms of maladaptive behaviour were poor work attendance and an inability to cope with the workload, leading to a failure to complete work-related tasks. The study’s findings align with research that determined psychiatric nurses often experience burnout and frustration. Their experience is related to stressful situations following an encounter with violent patients (Joubert & Bhagwan 2018:54; Ramalisa, Du Plessis & Koen 2018:2). It is further confirmed in the study conducted by Ramalisa et al. (2018:7) that the violence psychiatric nurses experience in the workplace affects their mental health status.

Similar to other studies (Bekelepi & Martin 2022:3; Harris et al. 2015:134), the findings of this study confirm that male psychiatric nurses exposed to violence perceive the environment as unsafe. This experience made them fearful when caring for state patients (Bekelepi & Martin 2022:4). Furthermore, Bekelepi and Martin (2022:5) reported that psychiatric nurses who experienced violence lived in fear, shock, anger and disbelief, leaving them feeling helpless. The psychiatric nurses exposed to violence in the latter study demonstrated intense emotional states of anger and feeling violated, resulting in lengthy periods of absence from work and taking time off to deal with the effects of the incident. Similarly, the male psychiatric nurses in this study became demotivated to go to work and were frequently absent to avoid encounters with violent state patients. According to Pelto-Piri, Warg and Kjellin (2020:6), psychiatric nurses who experienced violence in psychiatric inpatient settings found it challenging to cope. They considered such acts of violence very stressful psychologically, affecting their enthusiasm for work.

Ultimately, the violence perpetrated in maximum-security forensic units affected the male psychiatric nurses who were left in the units because of the absenteeism rate. They had to manage increased workloads when their colleagues were absent, which increased the likelihood of psychological stress. Hasan and Tumah (2019:157) also reported that psychiatric nurses who were left to care for patients when others were absent assumed additional tasks, further exacerbating their workload.

Moreover, the lack of security personnel in maximum-security forensic units cannot be overlooked. As in this study, Mulaudzi et al. (2020:5) revealed that psychiatric nurses experienced a lack of security as an impediment to their duties, exposing them to high risks of violence. However, although the exposure to violence rendered their working environment unsafe, the male psychiatric nurses in this study learnt to accept it as part of their work lives. These findings support those reported by Foster, Cuzzillo and Furness (2018:342), who confirmed that psychiatric nurses accept violence as part of their working conditions. Interestingly, Itzhaki et al.’s (2018:4) findings also revealed that psychiatric nurses develop social tolerance towards violence as an integral component of their job.

The male psychiatric nurses in this study identified various measures to minimise the occurrence of violence. As a result, they assumed the role of security personnel to some extent to create a safe space for themselves and other state patients they were caring for. The male psychiatric nurses became more vigilant and had to enforce safety protocols when caring for state patients in the forensic units. Subsequently, continuous supervision of the state patients became central to their approach to care. This view is supported by Abela-Dimech, Johnston and Strudwick (2017:110), who remarked that psychiatric nurses develop standardised search protocols to prevent potentially dangerous items from entering psychiatric units. The Department of Health: Victoria (2021:NP) similarly suggests that psychiatric nurses should search patients’ belongings to ensure the safety of patients and staff in mental health institutions. In addition to the search, continuous supervision of the patients is recommended to create a safe environment for the nursing staff and other patients (O’Shea et al. 2019:23). The male psychiatric nurses in this study thus supervised state patients continuously to ensure safety was always maintained.

Psychiatric nursing and forensic nursing skills are vital when caring for violent people. The male psychiatric nurses in this study could use these skills to execute their duties while managing violence in the forensic units. However, they still possess no forensic nursing skills. According to Moss (2015:44), patients with mental illness, especially those who exhibit violence, require effective communication skills for their care and management. A study conducted in the Western Cape, South Africa, also concurs that psychiatric nursing skills are essential in managing violent patients (Bekelepi et al. 2015:161).

Moreover, Adeniyi and Puzi (2021:4) assert that psychiatric nurses should develop effective communication skills to promote verbal de-escalation, as they are typically on the front line when a patient becomes violent. The above statements reflect what male psychiatric nurses in this study practised to calm violent state patients. According to Itzhaki et al. (2018:4), interpersonal skills and behaviour management practices have had positive effects when psychiatric nurses intervene in violent situations.

## Limitations

This study only focused on male psychiatric nurses. Further research should include female psychiatric nurses working with female forensic patients to obtain an in-depth understanding of their experiences and compare them to those of male psychiatric nurses. This could be used to identify training needs that could be used to develop specialist knowledge and skills through the training and development of nurses working in such specialised areas. Furthermore, the research was conducted in one specific psychiatric hospital in Gauteng, South Africa. There is a need to conduct research in other psychiatric hospitals to compare the findings.

## Recommendations

The findings of this study are consistent with literature confirming that the experience of violence is traumatic and thus requires set skills to cope with the psychological impact (Adeniyi & Puzi 2021:4; Barr et al. 2019:892; Bekelepi et al. 2015:161; Moss 2015:44; Itzhaki et al. 2018:4). The lack of such skills impacts on the ability of psychiatric nurses to cope with the psychological impact. Maximum-security forensic units are specialised care areas and therefore require that psychiatric nurses in maximum-security forensic units be trained. The promulgation of the new training regulations by the South African Nursing Council could see the birth of psychiatric nurses trained in forensic nursing as a specialised field. More needs to be done to identify male psychiatric nurses who will be trained in the specialised field of forensic nursing.

Psychiatric nurses in maximum-security forensic units play a pivotal role in state patients’ treatment and rehabilitation. Thus, measures must be implemented to provide ongoing support and ensure their psychological well-being. In addition, psychiatric and forensic skills should be enhanced among those managing violent patients. Recommendations made by Durcan et al. (2017:43) highlight the need to invest staff wellbeing, mentoring and training, more consistent education and training for mental health practitioners including psychiatric nurses who play a role in the care of those treated in maximum-security forensic units. While this study reflected that violence in maximum-security forensic units is inevitable, continuous supervision, adherence to safety protocols, creating a safe environment and advanced psychiatric nursing skills – including effective communication skills – assisted the psychiatric nurses to minimise the risk of exposure to violence. This highlights the need for ongoing psychological support that did not come out clear in this study, skills training and collaboration with other team members.

## Conclusion

The male psychiatric nurses desired to work in forensic units, but their experience was described as traumatic and negatively impacted them. Violence could affect their mental health. As such, the study created awareness of the impact of violence in forensic units. The male psychiatric nurses in this study described survival strategies they used to cope with the impact. Psychological support and skills development exposing them to advanced psychiatric nursing skills as well as forensic nursing training could benefit male psychiatric nurses working in maximum-security forensic units to effectively manage violence. The male psychiatric nurses could benefit if collaboration with other multidisciplinary team members is strengthened in order to reduce the incident of violence in forensic units.
